# Transformer Learning in Sequence‐Based Drug Design Depends on Compound Memorization and Similarity of Sequence‐Compound Pairs

**DOI:** 10.1002/minf.70016

**Published:** 2026-01-08

**Authors:** Jürgen Bajorath

**Affiliations:** ^1^ Department of Life Science Informatics Bonn‐Aachen International Center for Information Technology Rheinische Friedrich‐Wilhelms‐Universität Bonn Bonn Germany; ^2^ Lamarr Institute for Machine Learning and Artificial Intelligence Bonn‐Aachen International Center for Information Technology Rheinische Friedrich‐Wilhelms‐Universität Bonn Bonn Germany

**Keywords:** chemical language models, encoder‐decoder architecture, explainable artificial intelligence, learning characteristics, sequence‐based compound design, transformers

## Abstract

Chemical language models (CLMs), particularly encoder‐decoder transformers, have advanced generative molecular design. Transformer CLMs are able to learn a variety of molecular mappings for compound design that can be conditioned using context‐dependent rules. However, their black‐box nature complicates the interpretation of predictions. Current analysis methods mostly focus on attention weights of token relationships or attention flow in encoder and decoder modules and cannot explain predictions at the molecular level. Sequence‐based compound design was used as a model system to investigate transformer learning characteristics through systematic control calculations involving modifications of protein sequences and sequence‐compound pairs. The analysis revealed that compound reproducibility depended on similarity relationships between training and test data and on compound memorization, while specific sequence information was not learned. These findings indicate that predictions of transformer CLMs are driven by memorization effects and statistical correlations rather than by learning specific chemical or biological information. Understanding this learning behavior aids in avoiding over‐interpretation of model outputs and informs the appropriate application of transformer‐based CLMs in molecular design.

## Graphical abstract

Transformer model. Shown is a schematic representation of an encoder‐decoder transformer trained for protein sequence‐based compound design.

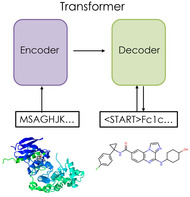

## 1 Introduction

This Mini‐Review summarizes the content of a lecture at the 9^th^ Autumn School on Chemoinformatics in Nara (http://www‐dsc.naist.jp/dsc_naist/en/autumn‐school‐2025/) and is a contribution to the corresponding article collection.

Chemical language models (CLMs) provide many opportunities for molecular design [1, 2, 3]. A major attraction is their generative modeling capacity. Transformer networks [4] with their characteristic attention and self‐attention mechanism [4, 5] were responsible for major advances in natural language processing and have also become a preferred architecture for CLMs [3, 6]. Transformers represent the framework of many task‐specific generative models used in biology, chemistry, and other scientific fields as well as of large language models. Different architectural variants of transformers are available consisting of encoder‐decoder combinations, only encoders, or only decoders [7]. Most CLMs are encoder‐decoder transformers that learn mappings of textual representations of molecules such as Simplified Molecular Input Line Entry System (SMILES) [8] or other strings. These mappings can be conditioned on context‐dependent rules such as molecular property constraints [2, 3]. For transformer learning, text‐based molecular representations are encoded as sequences of tokens. Transformer encoder and decoder modules include multihead attention and fully connected feed‐forward neural network layers [4, 7]. In a multihead (self‐)attention layer, several attention functions act in parallel on different sequence segments to generate attention vectors. Attention weights are derived to quantify the importance of pairwise token relationships in sequences or across input and output sequences [6]. As long as text‐based molecular representations can be appropriately tokenized, transformer CLMs are able to learn a variety of molecular mappings for generative modeling. Current applications include off‐the‐beaten path design tasks that would be difficult or impossible to address with other computational methods. An exemplary task is sequence‐based compound design, which requires learning of target protein sequence‐to‐compound mappings, as discussed in the next section.

## 2 Sequence‐Based Compound Design

Protein sequence‐based compound design attempts to generate new active compounds using protein sequences as input. One may well argue that sequence‐based compound design lacks a strong scientific foundation. In structure‐based drug design, complementarity between a given ligand binding site and candidate compounds is computationally explored and exploited. By contrast, it is difficult, if not impossible, to define sound scientific criteria for establishing relationships between sequence data and compound structures and for developing corresponding design strategies. Two decades ago, first attempts were made to predict drug‐target interactions based on combined representations of target sequences and compound structures [9, 10, 11], mostly by classifying true and false protein‐ligand associations with neural networks or support vector machine (SVM) models. In 2011, a similar approach was applied to virtually screen for active compounds based on target sequences using an SVM model [12], representing the first (and probably only) attempt to predict active compounds from protein sequences using conventional machine learning methods. With the advent of CLMs, sequence‐based compound design has been revisited. While no substantial progress was made over the years in deriving scientific principles underlying this approach, learning sequence‐to‐compound (input/output) mappings using CLMs and then generating new compounds based on input sequences became readily possible. Several recent studies have reported successful retrospective or prospective predictions of active compounds based on complete target sequences or sequence motifs of ligand binding regions using CLMs [13, 14, 15, 16, 17]. In one of the first applications, a transformer was trained to learn mappings of complete protein sequences to corresponding active compounds represented as SMILES strings and predict new compounds based on input test sequences [13]. Furthermore, an Lmser network‐based transformer variant with multihead cross attention blocks was implemented to map complete protein sequences to active compounds [14]. The encoder processed the protein sequence and the latent space was decoded into compound strings with the aid of Monte Carlo tree searching [14]. Both studies used protein‐ligand docking scores to prioritize newly generated candidate compounds [13, 14]. An encoder‐decoder transformer was derived to learn mappings of sequence motifs of the ATP and inhibitor binding site in protein kinases to active compounds [15]. In this study, the ability of the model to exactly reproduce ATP site‐directed inhibitors of different kinases excluded from training was used as an evaluation criterion [15]. Another transformer model processed sequence embeddings and compound atom embeddings generated with a protein language model and a graph convolutional neural network, respectively, to predict new active compounds [16]. The transformer was shown to generalize across different targets and compound classes and interpreted to learn information relevant for ligand binding. Selected candidate compounds were experimentally validated [16]. In another study, a protein language model for generating sequence embeddings was combined with a conditional encoder‐decoder transformer to predict potent compounds for given targets [17]. Therefore, the transformer was pretrained to learn mappings of concatenated protein sequence and compound potency value embeddings to corresponding compounds. Then, the pretrained transformer was fine‐tuned on individual compound activity classes not encountered during training and evaluated on structurally diverse compound test sets [17].

## 3 Explainable Artificial Intelligence for Transformers

The black‐box nature of many machine learning and all deep learning models has given rise to increasing interest in explainable artificial intelligence (XAI) [18, 19]. This AI subdiscipline aims to develop concepts and methods to computationally explain inner workings and predictions of machine learning models and make these explanations accessible to human reasoning [18, 19]. The inability to explain and interpret predictions of machine learning models is not only scientifically unsatisfactory but also limits the impact of machine learning in interdisciplinary research. Transformer models are particularly difficult to explain, especially if applied to generative design tasks outside natural language processing [19, 20]. Currently available XAI approaches for transformers largely focus on the analysis of attention weights [20, 21, 22], attention flow across different layers [20, 23], or weight gradients [24]. For token pairs, attention weights are often visualized in heatmaps or bipartite network representations (comparing two sequences) [20, 21, 22]. For visualizing attention flow, attention layers are also represented as token‐based networks in which edges represent weights [20, 23]. In addition to attention weight‐based methods, a variant of the Shapley additive explanations (SHAP) method for approximating Shapley values as feature attribution scores [25, 26] has been introduced for encoder‐only transformers, termed TransSHAP [27]. This SHAP variant is applicable to quantify input token importance for text classification or next‐token prediction [27], but not to other generative design tasks. For transformer CLMs, there currently is no XAI approach available to explain generative design at the molecular level of detail. However, as discussed in the following, learning characteristics of transformer CLMs can also be explored using suitable model systems such as sequence‐based compound design.

## 4 Analyzing Transformer CLM Learning

We have selected sequence‐based compound design as a model system for exploring the learning behavior of a transformer CLM in detail. The choice of this model system was motivated by the possibility of modifying protein sequences and sequence‐compound pairs in a variety of ways, thereby providing the basis for systematic control calculations.

For the analysis, a transformer with the original encoder‐decoder architecture [4] was pretrained using ∼157,000 sequence‐compound pairs covering 1419 unique target proteins. For learning mappings of sequences to compounds, positional residue‐based sequence encoding and atom‐based encoding of canonical SMILES strings was applied. Pretrained models were evaluated on hold‐out sets of 30% of the original data [28].

### 4.1 Critical Importance of Similarity Relationships

**FIGURE 1:**
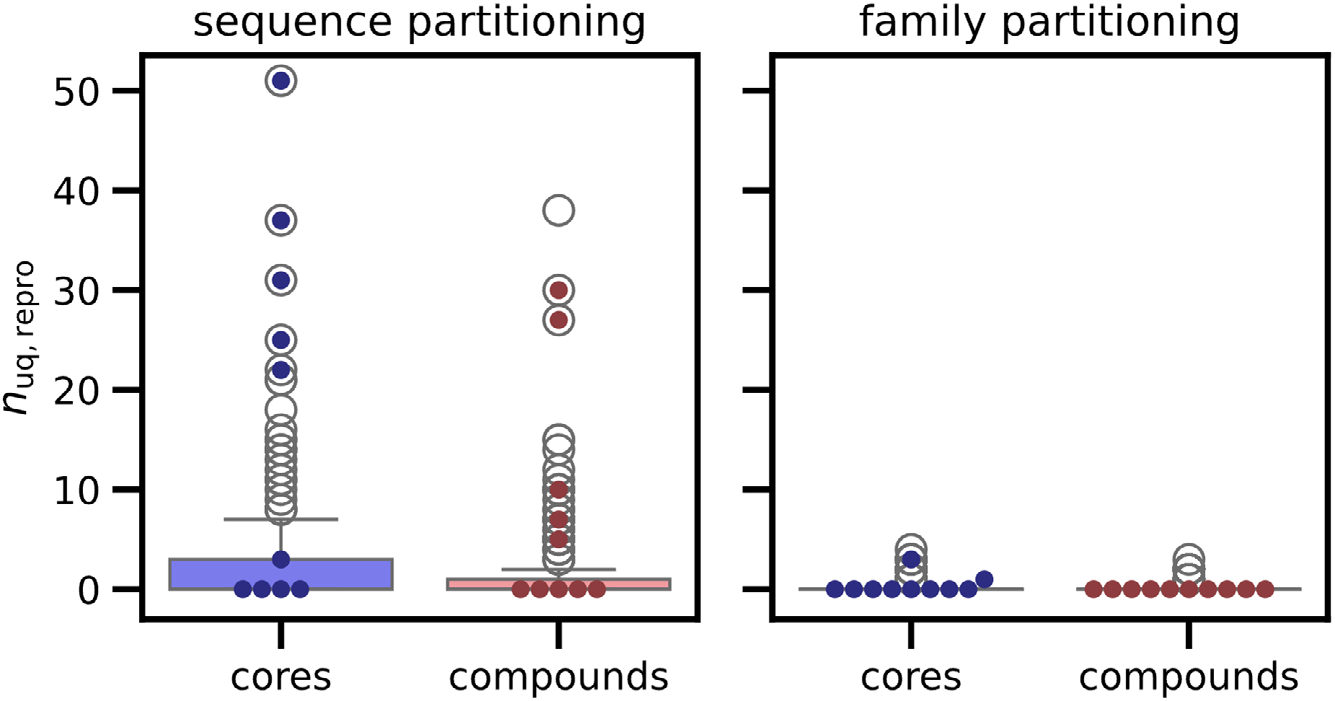
Reproduced unique active compounds and cores. Boxplots (box: 1st quartile, median, 3rd quartile; whiskers: +/−1.5 × interquartile range) show the distribution of unique correctly reproduced (*n*_uq,repro_) test compounds and cores for test sequences with more than 20 available compounds and pretrained transformer models based on sequence and family partitioning, respectively. Dots represent the 10 test sequences with the largest number of associated compounds. The figure was adopted from an open‐access publication of the author and modified [28].

Pretraining was performed based on two data partitioning schemes. In sequence‐based partitioning, sequences and associated compounds were randomly divided into training and test set pairs. Accordingly, the training and test sets contained pairs of similar sequences and compounds from the same protein families. Alternatively, in family‐based partitioning, sequences and compounds of entire protein families were either assigned to the training or test set. Therefore, training and test sets did not contain closely related sequence‐compound pairs. The two pretrained models were evaluated by determining exactly reproduced active compounds and core structures for test sequences. Figure 1 shows the distribution of unique exactly reproduced compounds and cores for test sequences with more than 20 available compounds. The sequence‐based pretrained model generally reproduced test compounds and cores, with a mean of two to three compounds and cores per sequence. By contrast, the family‐model essentially failed to reproduce compound and cores.

Thus, reproducibility strongly depended on the presence of similar sequence‐compound pairs in training and test data resulting from random data partitioning that were reduced or eliminated by family‐based partitioning.

### 4.2 Compound Memorization Effects

**FIGURE 2:**
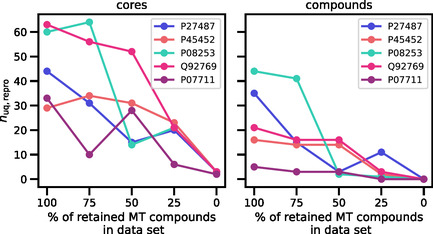
Influence of multitarget compounds on reproducibility. For five test sequences (with UniProt [29] accession numbers) with five to more than 40 active test compounds reproduced with the original sequence‐based pretrained model (100% MT compounds), the number of reproduced unique (*n*_uq,repro_) cores (left) and compounds (right) is reported for sequence‐based model variants pretrained in the presence of decreasing numbers of MT compounds. The figure was adopted from an open‐access publication of the author [28].

Approximately 30% of all source compounds were active against more than one target protein, with a mean of approx. three proteins (sequences) per multitarget (MT) compound. As a consequence, MT compounds paired with different sequences were present in the training and test data sets. To assess the influence of MT compounds on reproducibility, different versions of the sequence‐based pretrained model were generated and evaluated following stepwise elimination of individual sequence‐MT compound pairs until only one pair remained for each MT compound. Accordingly, MT compounds were iteratively converted into single‐target compounds that could only be present in either training or test data. Figure 2 shows that the number of reproduced active test compounds and core structures decreased with decreasing numbers of retained MT compounds.

In the absence of MT compounds, no test compounds and only few cores were exactly reproduced. Thus, compound memorization played a key role for reproducibility. Accordingly, the models memorized MT compounds encountered during pretraining and regenerated these compounds for test sequences that were similar to the training sequences paired with these MT compounds.

### 4.3 Fine‐Tuning and Systematic Sequence Variations

The original sequence‐based pretrained model was separately fine‐tuned on two pharmaceutical target protein families including the Ser/Thr protein kinase (CMGC) family (50 unique targets) and the G‐protein coupled receptor (GPCR) 1 family (148 unique targets). The ensuing models were tested on hold‐out sets of 30% of the sequences. As expected, fine‐tuning consistently increased the compound reproducibility for sequences of these families compared to the pretrained model [28].

Then, different versions of fine‐tuned CMGC and GPCR 1 family models were generated following systematic sequence modifications including masking of specific sequence motifs and cumulative sequence randomization. Signature motifs of the CMGC and GPCR1 family as well as the ATP/inhibitor binding region of the CMGC family were retrieved from PROSITE [30] and randomized. As a control, for each motif, the same number of residues was randomized at randomly selected positions throughout the sequence. Compared to the control, masking of sequence motifs did not lead to statistically significant changes in compound or core reproducibility. Thus, the fine‐tuned models did not appear to learn sequence information characteristic of these protein families or relevant for inhibitor binding [28].

**FIGURE 3:**
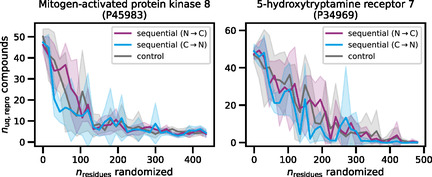
Compound reproducibility for models after cumulative sequence randomization. For an exemplary CMGC kinase and GPCR, the number of unique exactly reproduced test compounds (*n*_uq,repro_ compounds) is reported for variants of the fine‐tuned models following cumulative sequence randomization (*n*_residues_ randomized). Control calculations iteratively randomized the same number of residues at randomly selected positions across the entire sequences. The lines report the mean of five independent trials for each randomization step and the shaded areas report the standard deviations. The figure was adopted from an open‐access publication of the author and modified [28].

In cumulative sequence randomization,15‐residue segments in test sequences were iteratively randomized from the N‐ to the C‐terminus or in opposite direction until the sequences were fully randomized. As a control, at each step, the same number of residues was randomized at randomly selected positions throughout the sequence. Figure 3 shows representative test results for both families.

For increasing numbers of randomized residues, reproducibility decreased, as one would anticipate. However, the effects of cumulative randomization were direction‐ and also position‐independent, as revealed by the control calculations. Maintaining ∼50–60% of native sequences was sufficient for reproducing test compounds, regardless of the randomized residues. For retaining up to 50% of the original reproducibility, randomization of ∼150–200 residues was tolerated for both protein families. Surprisingly, for some fully randomized kinase (but not GPCR) sequences, including the one of the exemplary kinase shown in Figure 3, up to five test compounds were still reproduced. However, when exploring this apparent artifact, all reproduced test compounds turned out to be highly promiscuous kinase inhibitors, hence further supporting the presence of compound memorization effects, as discussed above.

### 4.4 Learning Characteristics

CLM variants based on alternative and modified sequence‐compound pair distributions revealed that similarity between sequence‐compound pairs in training and test sets as well as compound memorization were critical for compound reproducibility. Furthermore, other model variants based on systematic manipulation of input sequences indicated that the transformers did not learn specific sequence information. By contrast, randomization of a substantial proportion of residues was tolerated. Many combinations of noncontiguous sequence fragments were sufficient for learning, as long as about half of the original residues were retained. Hence, sequence‐based compound design using transformers was determined by learning similarities between paired sequences and compounds and by deriving statistical sequence‐compound correlations.

## 5 Discussion and Conclusions

CLMs have opened the door to a variety of generative molecular design tasks. However, these models are black boxes, which limits their acceptance for experimental applications in chemistry. Current XAI approaches predominantly concentrate on the analysis of attention weights and attention flow in natural language processing or image analysis. For transformer CLMs, methods to explain and interpret predictions at the molecular level of detail are currently unavailable. However, learning characteristics of transformers CLMs can also be explored based on carefully planned machine learning control calculations. To this end, we have used sequence‐based compound design as a test system to better understand transformer learning in molecular design. Addressing this prediction task is difficult, if not impossible using standard machine learning methods, but straightforward using CLMs by learning sequence‐to‐compounds mappings. Our analysis involved many control calculations after modifying sequences and sequence‐compound pairs and revealed that similarity of sequence‐compound pairs in training and test sets and compound memorization were critical for compound reproducibility. Furthermore, transformer model variants did not learn specific sequence information, and even subsets of fragmented sequences were sufficient for deriving statistical correlations underlying predictions. Thus, the predictions were primarily statistically driven by the detection of similarity relationships and sequence‐compound correlations. Importantly, the transformer CLMs were not required to learn underlying chemistry. These findings currently apply specifically to sequence‐based compound design using transformers and cannot be generalized for molecular design. It remains to be determined if information relevant for ligand binding is learned in other applications. However, the author anticipates that purely statistical correlations dominate many chemical and biological predictions.

Notably, the ability of transformers to memorize compounds and detect sequence‐structure correlations is in principle not an undesirable feature or alternative to learning chemical and/or biological relationships, if attractive candidate molecules can be predicted. Going forward, an important question will be how to best exploit memorization ability, which depends on the applicability domain of the models. Importantly, understanding transformer learning behavior and the purely statistical nature of their predictions, at least in sequence‐based compound design, helps to avoid model over‐interpretation and the promotion of Clever Hans predictors. This is of critical relevance for a realistic scientific assessment of generative molecular design using transformers.

It should also be taken into consideration that the scientific rationale underlying sequence‐based drug design arguably is not very strong in the first place and much weaker than the guiding principles and underlying rationale of structure‐based drug design. As a consequence, structure‐based approaches have dominated molecular design taking protein information into account over decades. One might well ask how a model is supposed to extract knowledge from global sequence information that is relevant for specific ligand binding, if it is questionable whether such knowledge even exists. On the other hand, equivariant transformers and diffusion models are increasingly applied to structure‐based design tasks and exploring their learning characteristics might provide—in the presence of a strong scientific rationale—deeper insights into the potential of such models to learn scientifically relevant information for predicting protein‐ligand interactions, beyond statistical correlations. This remains to be investigated. Regardless, numerous applications of machine learning in the field of molecular design have shown that recognizable molecular similarity relationships play a crucial role for successful predictions—and this also applies to the transformer models we have analyzed thus far. Their strong memorization ability for sequences and chemical structures further enhances the capacity to learn statistical correlations and detect similarity patterns. This might lead to compelling predictions in molecular design, even if the underlying chemistry or physics is not learned. Thus, from this perspective, the transformer learning features uncovered in sequence‐based compound design, regardless of whether they might be observed more generally, are in themselves neither negative nor positive. However, they must be placed into an appropriate scientific context to judge predictions and avoid Clever Hans effects.

## Conflicts of Interest

The author declares no conflicts of interest.

## Data Availability

The author has nothing to report.
